# Interstitial lactate, lactate/pyruvate and glucose in rat muscle before, during and in the recovery from global hypoxia

**DOI:** 10.1186/s13028-014-0072-0

**Published:** 2014-11-13

**Authors:** Norbert Zoremba, Aleš Homola, Rolf Rossaint, Eva Syková

**Affiliations:** Department of Anaesthesiology, University Hospital RWTH Aachen, Pauwelsstrasse 30, D-52074 Aachen, Germany; Department of Neuroscience, Charles University, 2nd Medical Faculty, V Úvalu 84, 150 06 Prague, Czech Republic; Institute of Experimental Medicine, Academy of Sciences of Czech Republic, Vídeňská 1083, 142 20 Prague, Czech Republic

**Keywords:** Hypoxia, Microdialysis, Muscle, Recovery, Lactate, Glucose, Metabolism

## Abstract

**Background:**

Hypoxia results in an imbalance between oxygen supply and oxygen consumption. This study utilized microdialysis to monitor changes in the energy-related metabolites lactate, pyruvate and glucose in rat muscle before, during and after 30 minutes of transient global hypoxia. Hypoxia was induced in anaesthetised rats by reducing inspired oxygen to 6% O_2_ in nitrogen.

**Results:**

Basal values for lactate, the lactate/pyruvate ratio and glucose were 0.72 ± 0.04 mmol/l, 10.03 ± 1.16 and 3.55 ± 0.19 mmol/l (n = 10), respectively. Significant increases in lactate and the lactate/pyruvate ratio were found in the muscle after the induction of hypoxia. Maximum values of 2.26 ± 0.37 mmol/l for lactate were reached during early reperfusion, while the lactate/pyruvate ratio reached maximum values of 35.84 ± 7.81 at the end of hypoxia. Following recovery to ventilation with air, extracellular lactate levels and the lactate/pyruvate ratio returned to control levels within 30–40 minutes. Extracellular glucose levels showed no significant difference between hypoxia and control experiments.

**Conclusions:**

In our study, the complete post-hypoxic recovery of metabolite levels suggests that metabolic enzymes of the skeletal muscle and their related cellular components may be able to tolerate severe hypoxic periods without prolonged damage. The consumption of glucose in the muscle in relation to its delivery seems to be unaffected.

## Background

The skeletal muscles are an elaborate energy production and consumption system that influences the whole body’s energy metabolism [1]. The metabolism of skeletal muscle is strictly regulated by substrate availability, the presence of oxygen and energy demands, which in turn also regulate muscle protein metabolism and cell size [2]. Hypoxia causes a deficiency in cellular oxygen supply and may lead to tissue damage, an inflammatory response and organ dysfunction [3]. This imbalance between oxygen consumption and supply initiates changes in extracellular metabolite and substrate levels, due to their release or uptake by cells to and from the extracellular space (ECS). Microdialysis is a highly sensitive technique used to monitor extracellular metabolite and substrate levels and allows the measurement of regional metabolic tissue concentrations [4].

In clinical practice lactate has been interpreted as a marker of anaerobic metabolism, if an imbalance between tissue O_2_ supply and demand occurs. Lactate accumulates as the end product of glycolysis when oxidative phoshorylation, as well as the tricarboxylic acid cycle are reduced [5-8]. The isolated view of lactate as a marker for anaerobic metabolism could be misleading. For the detection of anaerobic metabolism, the simultaneous determination of pyruvate levels is necessary, as pyruvate is reduced to lactate by lactate-dehydrogenase under anaerobic conditions. By examining the ratio between lactate and pyruvate (L/P ratio) the degree of aerobic/anaerobic metabolism can be specified, reflecting the cytosolic ratio of the reduced/oxidized forms of NAD. Therefore, the L/P ratio is a more reliable parameter for estimating the energy state of the cells [8]. In addition to the measurement of metabolic end products, the extracellular glucose level is an important factor in substrate equilibration. The glucose concentration in the ECS reflects the balance between the supply from blood and utilisation by cells [9].

The aim of our study was to evaluate the changes in the energy-related metabolites lactate and pyruvate as well as in the substrate glucose before, during and after global hypoxia in the skeletal muscle using in *vivo* microdialysis. These findings may help to describe the effects of hypoxia and reoxygenation on muscular aerobic and anaerobic metabolism and substrate levels.

## Methods

### Animal preparation and experimental protocol

Adult male Wistar rats (300–350 g and aged approximately 12–15 weeks), obtained from the same breeder (Velaz, s.r.o, Prague, Czech Republic), were housed in groups of 4 animals per cage before the experiment in breeding cages T IV (Velaz, s.r.o, Prague, Czech Republic). The cages was provided with spruce bedding (Lignocel® 3-4S, Rettenmaier und Söhne, Rosenberg, Germany), and no further enrichment were provided. The rats were housed in a 12:12 hour light:dark cycle with lights turning on at 6 o’clock in the morning. The room temperature was maintained at 22°C. Food (Altromin 1314 TPF standard diet, Altromin Spezialfutter GmbH & Co KG, Lage, Germany) and tapwater was provided ad libitum. The animals were anaesthetised by an intraperitoneal injection of urethane (1.5 g/kg body weight, Sigma-Aldrich Chemie GmbH, Seelze, Germany). Before the beginning of the surgical procedures, the depth of anaesthesia was checked by testing the corneal reflexes. If necessary, an additional 100 mg of urethane were injected intraperitoneally. To avoid any ventilatory interactions due to spontaneous breathing action, the animals were relaxed with pancuroniumbromide (0.4 mg/kg body weight, Pavulon, Organon, Netherlands), intubated and connected to a ventilator (CIV 101, Columbus Instruments, Columbus, Ohio, USA). The body temperature was maintained at 36–37°C by a heating pad. The sufficient depth of anaesthesia during the experiment was monitored by means of clinical signs (heart rate, lacrimation etc.). A small longitudinal incision of the skin was made above the back muscles to place the microdialysis probe. This device was slowly inserted into the musculus longissimus by an introducing canula. To avoid any possible influence of probe insertion tissue trauma on the microdialysis results, measurements started one hour after the placement of the microdialysis probe. Before hypoxia the animals were ventilated with air. Hypoxia was induced for 30 minutes by reducing the inspiratory oxygen content to 6% in 94% nitrogen. After this hypoxic period the animals were ventilated with air again. The control animals were ventilated with air during the whole experiment. This study was performed on hypoxic (n = 10) and control animals (n = 5), which were randomly assigned to the two groups before starting the experiment. At the end of the experiment the animals were euthanized by intraperitoneal injection of urethane and potassium chloride.

All efforts were made to minimize animal suffering and to reduce the number of animals used. The experiments were carried out in accordance with the European Communities Council Directive of 24^th^ November 1986 (86/609/EEC) and approved by the local Institutional Animal Ethics Committee.

### Microdialysis

The measurement of extracellular metabolite and substrates levels by microdialysis is based on sampling fluid via a double-lumen probe with an integrated semipermeable membrane. At this membrane an equilibration of substances in the extracellular space and the perfusion fluid takes place by diffusion according to the concentration gradient. We used a double-lumen microdialysis probe with a membrane length of 10 mm, an outer diameter of 0.5 mm and a cut-off at 20.000 Dalton (CMA 20, CMA Microdialysis, Sweden). In order to maintain a constant dialysate flow, the microdialysis catheter was connected via low-volume FEP-tubing (1.2 μl/10 cm) to a precision infusion pump (CMA 102, CMA Microdialysis, Sweden). The microdialysis catheter was continuously perfused with a dialysate containing 147 mmol/l Na^+^, 4.0 mmol/l K^+^, 2.3 mmol/l Ca^2+^ and 155.6 mmol/l Cl^−^ (Perfusion fluid T1, CMA Microdialysis, Sweden) at a flow rate of 2 μl/min. It has been shown that probe insertion damage causes a transient increase in metabolite levels, which return to stable baseline values within a stabilisation period of 30–60 minutes after probe insertation [10]. After an equilibration time of 60 minutes, microdialysate samples were collected at 10 minute intervals and immediately frozen at −40°C until analyzed. Dialysate samples were analysed enzymatically with a CMA 600 Microdialysis Analyser (CMA Microdialysis, Sweden) for lactate, pyruvate and glucose.

The exchange of substances across the microdialysis membrane is limited by the total area of the membrane, the perfusion flow rate, the characteristics of the diffusing substance and the diffusion constant in the tissue surrounding the probe [4,11]. These boundaries are described by the recovery rate, which expresses the relation between the concentration of the substance in the microdialysis probe effluent and the concentration of the medium [12]. At the beginning and end of the experiment, the recovery rates for each probe were determined by proceeding with the perfusion in the same settings in a defined calibration solution. The calibration solution contained 2.50 mmol/l lactate, 250 μmol/l pyruvate and 5.55 mmol/l glucose (Calibrator A, CMA Microdialysis, Sweden). The concentrations of these calibration solutions were compared with the concentrations of the *in vitro* microdialysis samples, and the relative recovery for each substance was calculated. The measured experimental values were weighted by the relative recovery to estimate the *in vivo* extracellular concentration of the components in the immediate vicinity of the probes. All results are presented as weighted concentrations.

### Statistical analysis

The results of the experiments are expressed as the mean ± standard error of the mean (SEM). Statistical analysis of the differences within and between groups was performed using Student’s paired t-test (InStat, GraphPad Software, San Diego, USA). Values of *P* < 0.05 were considered significant. If a statistical significant difference was found a post hoc power analysis was made with α =0.05.

## Results

### Lactate and lactate/pyruvate ratio

**Figure 1:**
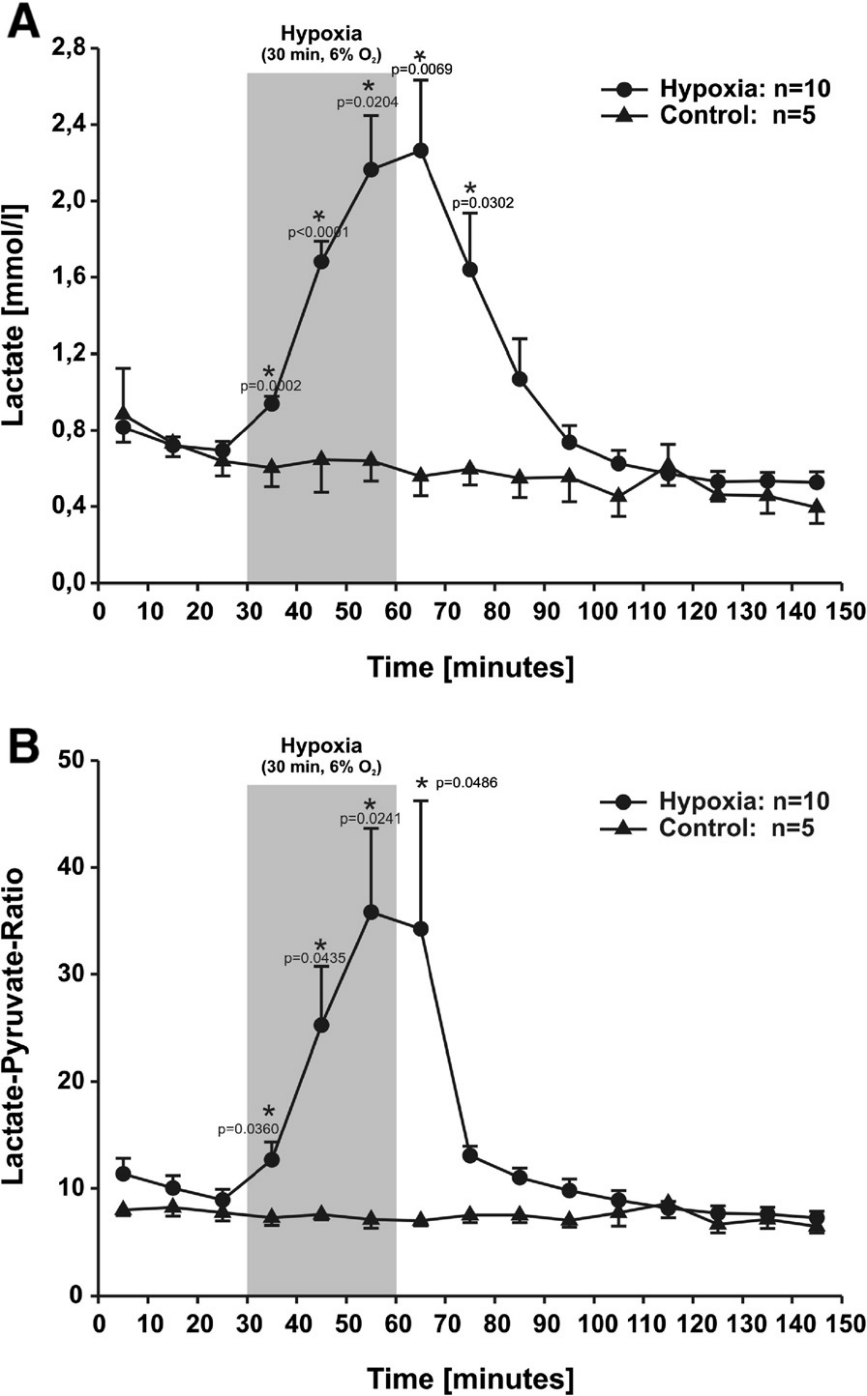
**Interstitial lactate levels (A) and the L/P ratio (B) before, during and after 30 minutes of hypoxia compared to controls.** After a stabilisation period, hypoxia was induced for 30 minutes by ventilating the animals with 6% oxygen in nitrogen (shaded area). Values are shown as mean ± SEM. The number of animals was n = 10 in the experimental and n = 5 in the control group. Statistically significant differences between the experimental and control groups were determined by Student’s t-test. Values of *P* < 0.05 are marked by “*”.

Before hypoxia, the basal muscular lactate levels remained stable at 0.72 ± 0.04 mmol/l (n = 10). Immediately after the onset of hypoxia (6% O_2_) a steep increase in extracellular lactate was measured. This rise continued throughout the hypoxic period of 30 minutes, reaching maximum values of 2.26 ± 0.37 mmol/l in the first measurement after the end of hypoxia. During reoxygenation with air, a recovery of the lactate level occurred, returning to the basal level within 40 minutes. In the control animals a basal lactate value of 0.73 ± 0.03 mmol/l (n = 5) was measured, which showed no significant changes during the measurement period. The difference between control and hypoxic animals was statistical significant (*P* <0.05) during hypoxia and early recovery (Figure 1A). In a post hoc power analysis a statistical power >80% was calculated. To recognize the switch to an anaerobic pathway, we calculated the lactate/pyruvate ratio (L/P ratio). In animals subjected to hypoxia a significant increase in the L/P ratio was seen from 10.03 ± 1.16 during baseline registration to 35.84 ± 7.81 (n = 10) at the end of the hypoxic period (Figure 1B). These changes in the L/P ratio produced a statistical power >80%. In the reoxygenation period the L/P ratio normalised, reaching baseline levels of 11.01 ± 0.88 within 30 minutes and remaining at this level until the end of the measurement period. In the control animals a basal L/P ratio of 8.22 ± 0.81 (n = 5) was registered. There were no significant changes in the L/P observed during the entire observation period.

### Glucose

**Figure 2:**
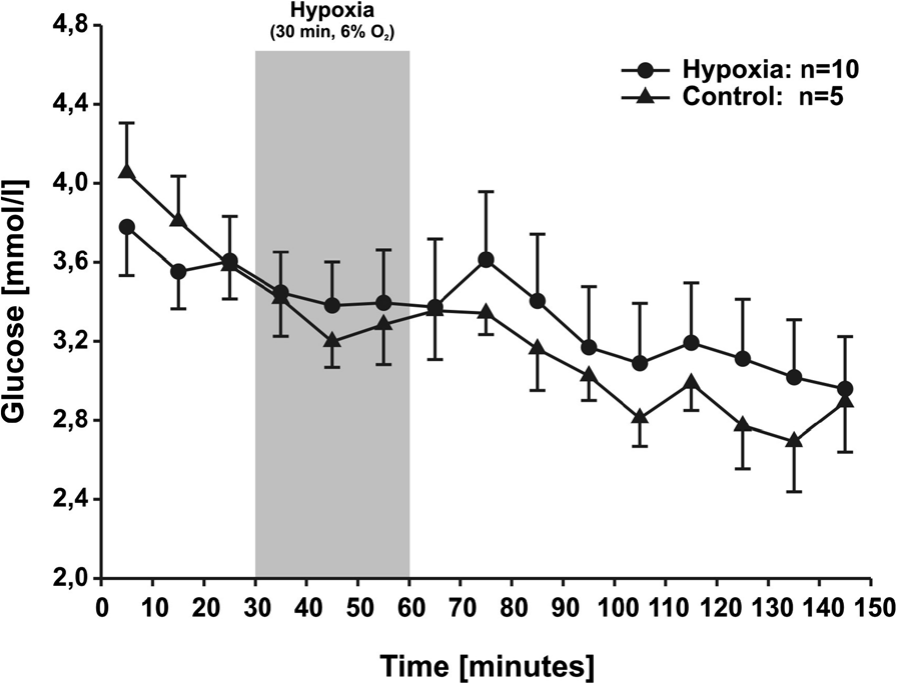
**Interstitial muscular glucose levels before, during and after 30 minutes of hypoxia compared to controls.** The duration of hypoxia is marked by a shaded area. Values are shown as mean ± SEM, and the number of animals was n = 10 in the hypoxic and n = 5 in the control measurements. No statistically significant differences were found between the groups.

Before the induction of hypoxia, stable basal glucose levels of 3.55 ± 0.19 mmol/l (n = 10) were found in the hypoxia group and 3.81 ± 0.22 mmol/l (n = 5) in the control group. During the whole experiment no significant differences were found between the two groups. At the end of the experiment a glucose level of 2.96 ± 0.27 mmol/l was measured in the hypoxia group. In the control group the final glucose level was 2.89 ± 0.25 mmol/l. The time course of the extracellular glucose levels in both groups is shown in Figure 2.

## Discussion

The aim of this study was to evaluate the changes in extracellular muscular lactate, the lactate/pyruvate (L/P) ratio and glucose levels during a hypoxic (6% O_2_) period of 30 minutes duration and subsequent reoxygenation. Microdialysis enables obtaining local information on energy metabolism in vivo in any tissue by introducing a probe directly into the region of interest [10]. An inspiratory oxygen content of 6% is the lowest possible level that can be used for this experimental model, as lower levels result in cardiac arrhythmia and cardiac failure. To the best of our knowledge no other evaluations of lactate, lactate/pyruvate ratio and glucose in the muscle were undertaken in an experimental setting with such a low hypoxic level. The possible influences of other organs were taken into consideration by choosing the rat as the experimental model animal.

In our experiments the onset of hypoxia was accompanied by a significant increase in muscular lactate levels and the L/P ratio. This increase in lactate levels and the lactate/pyruvate ratio is similar to changes observed in muscle that were reported in previous studies during hypoxia with 10% O_2_ [13,14]. Hypoxia leads to an increase in production of lactic acid due to a switch from aerobic to anaerobic metabolism. In aqueous solutions lactic acid dissociates almost completely to lactate and H^+^ [15]. An increase has also been found in different tissues as well as in the blood, accompanied by severe acidosis [14,16]. Severe acidosis may lead to possible disturbances in intra/extracellular ion distribution. The liberation of lactate may be much faster than its removal due to sufficient circulation. Additionally this increase in lactate level can be worsened by decreasing, hypoxia-induced hepatic lactate clearance [15]. Following hypoxia a fall in cellular ATP stimulates the activity of phosphofructokinase and an increase in glycolysis occurs. Nicotinamide adenine dinucleotide (NAD^+^), from the conversion of pyruvate to lactate, is required to support this increase in glycolysis [15]. Furthermore, an increase of glycolysis can be additionally amplified by endogenous secretion of catecholamines, due to stress during hypoxia.

During the reoxygenation period the elevated lactate levels recovered to prehypoxic values. This normalisation of extracellular muscular lactate levels could be caused by its release into the blood and consumption by the liver or by its uptake and consumption in the muscle. It was shown that blood levels during hypoxia are much higher than in the muscle and therefore the skeletal muscles act as lactate consumers even in the hypoxic state [13]. Muscles are also able to oxidize lactate, especially in oxidative fibers [17,18]. Thus, it could be hypothesized that during reoxygenation, the interstitial lactate was at least in part consumed in the muscle. In addition to the lactate level, the tissue L/P ratio is an excellent marker of anaerobic metabolism as it correlated closely with the redox potential [8]. In our study we found a steep increase in the L/P ratio starting immediately after the onset of hypoxia. This increase reflects the switch of the cytosolic redox condition from aerobic to anaerobic glycolysis. In the recovery from hypoxia a steep decrease in the L/P ratio indicates the return to aerobic conditions. These results indicate that energy-related cellular components (e.g. mitochondria) and their related metabolic enzymes may tolerate such hypoxic periods without any serious damage.

The extracellular glucose level is maintained by a balance between supply from the blood and utilisation by cells. During hypoxia no significant difference in muscular glucose levels between the hypoxia and control groups was found. It has been shown that during hypoxia the mean arterial blood pressure decreases about 50% from the prehypoxic values [14]. In the cerebral cortex, a tissue with high metabolism, reduction in arterial perfusion pressure during hypoxia results in a decrease in glucose levels and normalisation during the recovery [16]. It could be hypothesized that in skeletal muscle, despite a reduction in systemic blood pressure, a sufficient glucose supply is present. Therefore, the extracellular glucose concentration remained unaffected at the prehypoxic levels.

### Methodological considerations

In our experiments we used the microdialysis technique for determining regional metabolic tissue concentrations. This method is highly sensitive, but has some methodical limitations that must be addressed. The *in vivo* recovery of substances strongly depends on the surrounding tissue properties and can be influenced by various release, uptake and clearance processes [19]. The calculations of extracellular metabolite concentrations based on *in vitro* recovery may underestimate the interstitial concentrations [19-21]. However, the collected experimental data reflect the dynamic time course of extracellular microdialysate levels during and after hypoxia. These experiments represent the evaluation of energy-related metabolites and glucose levels during the lowest possible hypoxia levels in a full animal model. Further experiments with longer hypoxic periods are needed to describe in more detail the behaviour of skeletal muscles during and after hypoxic conditions.

## Conclusion

In conclusion, our findings suggest that 30 minutes of hypoxia influences the extracellular microenvironment of skeletal muscles and that the changes in energy-related metabolites caused by hypoxia recover to preischemic values within a few minutes. The energy-related cellular components (e.g. mitochondria) and their related metabolic enzymes may tolerate such a hypoxic period without any serious or prolonged damage. The glucose levels seem to be unaffected during and after hypoxia. It could be, that despite a possible reduction in systemic blood pressure, hypoxia does not affect sufficient supply to skeletal muscle.
